# The Pathophysiology and Treatment of Graft-*Versus*-Host Disease: Lessons Learnt From Animal Models

**DOI:** 10.3389/fimmu.2021.715424

**Published:** 2021-08-19

**Authors:** Takanori Teshima, Geoffrey R. Hill

**Affiliations:** ^1^Department of Hematology, Hokkaido University Faculty of Medicine, Sapporo, Japan; ^2^Clinical Research Division, Fred Hutchinson Cancer Research Center, Seattle, WA, United States; ^3^Division of Medical Oncology, The University of Washington, Seattle, WA, United States

**Keywords:** graft-*versus*-host disease, animal models, pathophysiology, history, treatment

## Abstract

Allogeneic hematopoietic cell transplantation (HCT) is a curative treatment for hematologic malignancies, bone marrow failure syndromes, and inherited immunodeficiencies and metabolic diseases. Graft-*versus*-host disease (GVHD) is the major life-threatening complication after allogeneic HCT. New insights into the pathophysiology of GVHD garnered from our understanding of the immunological pathways within animal models have been pivotal in driving new therapeutic paradigms in the clinic. Successful clinical translations include histocompatibility matching, GVHD prophylaxis using cyclosporine and methotrexate, posttransplant cyclophosphamide, and the use of broad kinase inhibitors that inhibit cytokine signaling (e.g. ruxolitinib). New approaches focus on naïve T cell depletion, targeted cytokine modulation and the inhibition of co-stimulation. This review highlights the use of animal transplantation models to guide new therapeutic principles.

## Early History

In 1956, two groups observed that mice exposed to lethal dose of total body irradiation (TBI) and administered allogeneic splenocytes survived for a shorter time than those transplanted with syngeneic splenocytes (1–3). The recipients of allogeneic cells exhibited diarrhea, weight loss, skin lesions, and died (1, 4). This syndrome was initially designated as “secondary disease”, which was later renamed as graft-*versus*-host disease (GVHD). In 1963, Mathé and colleagues reported the first case of a human allogeneic bone marrow transplantation (BMT) recipient that survived beyond a year. This patient had complete engraftment and the development of a lethal “secondary disease” was described (5). Subsequently, the clinical and pathological characteristics of GVHD was described (6). The outcomes for the initial 200 patients transplanted prior to 1967 were disparaging; all patients died of either graft failure, GVHD, infection, or leukemia relapse (7). These poor outcomes reflected a limited understanding of histocompatibility matching and the requirement for immune suppression after BMT to control GVHD (8).

**Figure 1:**
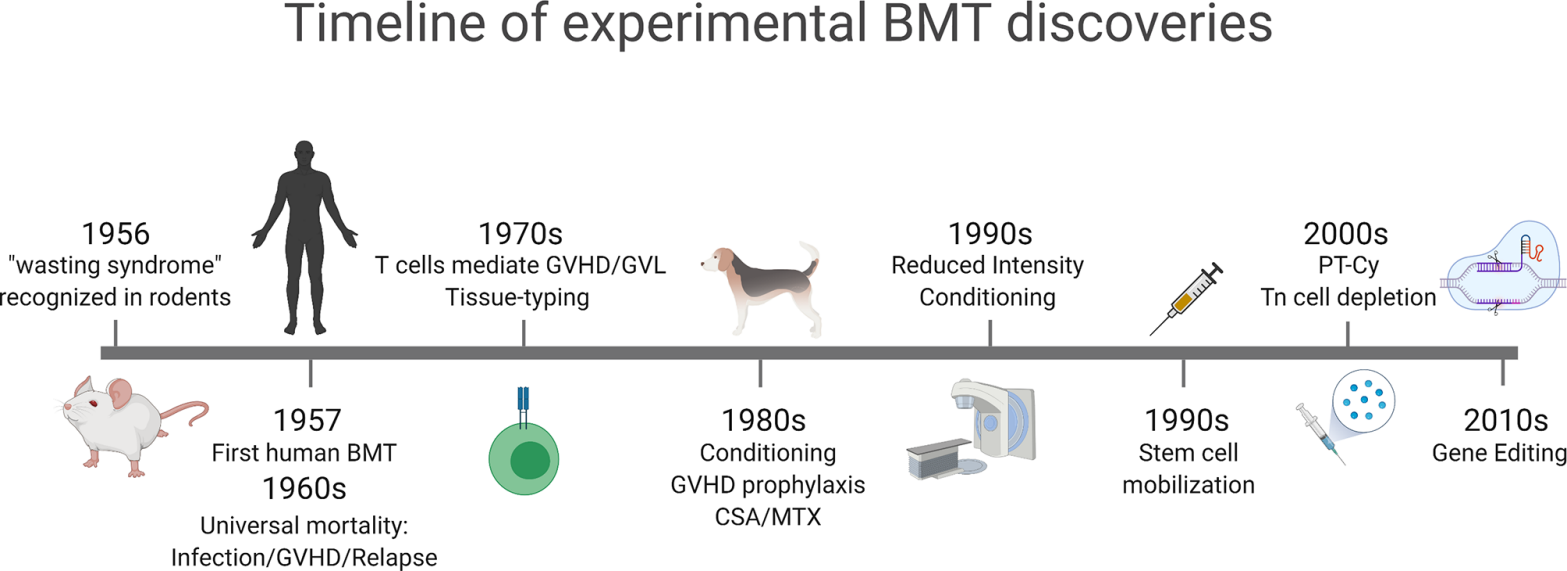
Timeline of major experimental concepts that have translated into clinical practice. GVHD was initially described as a wasting syndrome in transplanted mice in 1956. Early clinical bone marrow transplantation was associated with high mortality due to GVHD, infection and relapse. Recognition of T cells as the mediators of GVHD and GVL and initiating rudimentary tissue-typing. Conditioning and GVHD prophylaxis regimens were developed in dog models in the 1980s that led to reduced intensity and non-myeloablative conditioning in the 1990s. Stem cell mobilization following cytokine administration was developed in the early 1990s and gained widespread clinical translation. New approaches to GVHD prophylaxis, including post-transplant cyclophosphamide (PT-Cy) and naïve T (Tn) cell depletion developed in the 2000s and are increasingly utilized in the clinic. In the last decade the widespread use of gene editing, initially in T cells, has been widely translated to modulate GVHD and GVL. Figure generated with biorender.com.

Although many investigators lost their enthusiasm for BMT, several groups increasingly utilized animal models to gain a better understanding of the allogeneic barrier with regard to both GVHD and graft rejection. Studies of allogeneic BMT in Seattle using dog models in the 1980s provided the scientific groundwork for the field leading to the concepts of histocompatibility matching, conditioning regimens and pharmacological GVHD prophylaxis (9–13). These findings were soon translated to the clinic and successful clinical BMT was established (14), subsequently leading to E. Donnall Thomas being awarded the Nobel prize within Physiology or Medicine in 1990 (see **Figure 1** for a timeline).

For a detailed discussion of the various mouse models of GVHD currently available and the penetrance of disease therein, we refer the reader to some of the excellent reviews on this subject (15–17).

## Acute GVHD

### Donor T Cells

In the 1970’s and 1980’s, Korngold and Sprent performed extensive studies in a series of mouse models across MHC and/or minor histocompatibility antigen mismatches demonstrating the role of donor T cells in the etiology of GVHD. They showed that donor-derived T cells were causative of GVHD and identified T cell subsets (CD4 *versus* CD8) responsible for the induction of GVHD in each model (18–20). Following the observation that T cell depletion prevented GVHD in mice (18), clinical studies confirmed that GVHD also failed to develop following rigorous donor T cell depletion with CD34 positive selection of the donor inoculum and the administration of anti-thymocyte globulin (ATG); none developed GVHD even without posttransplant immunosuppression (21, 22).

Donor T cells exert graft-*versus*-leukemia (GVL) effects. In 1956, Barnes et al. reported that leukemia-bearing mice receiving allogeneic cells eventually died of GVHD without evidence of leukemia (2). Mathe et al. proposed a concept of GVL effect (23), which was soon demonstrated clinically (24–27). Importantly, pan T-cell depletion was shown to reduce GVHD at the expense of an increased risk of opportunistic infection and leukemia relapse (28). Shlomchik and colleagues refined our understanding of the subsets of mature T cells responsible for GVHD, demonstrating that naïve T cells rather than memory T cells played the major role in inducing GVHD in mice (29, 30). Early clinical trial data of naïve T cell-depleted PBSCT has shown promising results (31), but definitive randomized data is needed to confirm a role of naïve *versus* memory T cells in GVHD and GVL.

The predominant expansion of Th1/Tc1 and Th17/Tc17 cells in mice and the cytokines derived from these cells suggests that acute GVHD is primarily driven by Th1/Tc1- and Th17/Tc17-associated immune reactions (32–35). There is a crosstalk between GVHD and infection; GVHD-associated immunodeficiency, dysbiosis, and disruption of epithelial and mucosal barrier are risks for infections, while bacterial and viral infections are risks for GVHD by activating innate immunity (36). Neutrophils activated by translocation of intestinal bacteria can also accelerate GVHD early after BMT *via* tissue injury (34). Mechanistically, bacteria and virus-derived molecules behave as pathogen-associated molecular patterns (PAMPs) that accelerate allogeneic T cell responses. Candida colonization is a risk for acute GVHD and fluconazole prophylaxis is associated with reduction of severe acute GVHD (37, 38). In mouse models, fungal cell wall components such as sugar polymers, are recognized by macrophages and promote Th17 differentiation that exacerbate GVHD (39). These results highlight importance of infection prophylaxis in the control of GVHD.

Unfortunately, it is impossible to discern cause and effect from human microbiota studies which generate associations between bacterial taxa typically derived from 16s ribosomal sequencing and transplant outcomes (40). The use of shotgun sequencing allows for the imputation of various functional properties of bacterial species (e.g. the likely ability to generate various metabolites) which provide further granularity and allows hypothesis generation (41). Recently, gnotobiotic and/or antibiotic decolonized mice have allowed true cause and effect to be ascertained whilst permitting dissection of the mechanisms by which microbiota invoke GVHD, both at initiation and amplification phases of the disease (42, 43).

Regulatory T cells (Treg) defined by the transcription factor FoxP3 are pivotal for the maintenance of self-tolerance and the induction of tolerance after allogeneic hematopoietic stem cell transplantation (HCT). Depletion of CD25^+^ cells from the donor inoculum exacerbates acute GVHD and infusion of CD4^+^CD25^+^ Treg inhibits GVHD in mice (44–46). Mogamulizumab, anti-CCR4 antibody, eliminates Treg, in which CCR4 is highly expressed (47). Pretransplant administration of mogamulizumab is a risk for severe acute GVHD (48). Following the link between Treg and acute GVHD, early phase clinical studies of Treg infusion demonstrate safety of Treg infusion (49–54) but definitive data on efficacy is awaited. The use of epigenetic modifiers such as histone deacetylase inhibitors has been shown to attenuate acute GVHD and enhance regulatory T cell activity in preclinical systems (55). Promising activity in subsequent early phase clinical GVHD prophylaxis studies has also been seen (56). Additional immunomodulatory effects of these agents have recently been reviewed elsewhere (57).

GVHD prophylaxis using calcineurin inhibitors (cyclosporin or tacrolimus) reduce the expansion of effector T cells (Teff) by blocking IL-2 and prevent acute GVHD, but fail to reduce chronic GVHD (58–60). Calcineurin inhibitors regulate Teff at the expense of Treg inhibition and the major challenge of GVHD prophylaxis is to selectively control Teff, while preserving Treg. In addition, calcineurin inhibitors are not sufficient in isolation to control GVHD in HLA-mismatched HCT (61, 62). Alternative approaches have been explored in preclinical systems. A study in the early 1960’s showed that high-dose cyclophosphamide (Cy) prolonged murine skin allograft survival only when given shortly after transplant (63). Mayumi and Nomoto then continued studies to elucidate mechanisms of tolerance induction by post-transplant Cy (PTCy) in mice (64). Tolerogenic effects of PTCy were exerted through selective elimination of alloreactive T cells, while preserving bystander T cells and Treg (65–67). Subsequently, the Johns Hopkins group translated PTCy to the clinic and confirmed a low incidence of both acute and chronic GVHD, even after haploidentical HCT (68, 69). GVHD prophylaxis using PTCy is a standard of care in haploidentical HCT and also potentially in HLA-matched related and unrelated donor transplantation, either with bone marrow or peripheral blood stem cell sources (70, 71).

### Role of the Conditioning Regimen

In the setting of BMT, donor T cells are infused into recipients that have potentially experienced tissue injury by prior treatments of the underlying malignancy, infections, and more immediately, pre-transplant conditioning. The inflammatory environment invoked by these therapies predispose to a state of enhanced alloantigen-presentation. Johnson and Truitt demonstrated that delayed infusion of donor T cells induced less severe GVHD (72). The Ferrara group demonstrated that the conditioning regimen, particularly total body irradiation (TBI), induced proinflammatory cytokine secretion (e.g. IL-1 and TNF) and increased the severity of acute GVHD in animal models (73, 74). These studies demonstrated that GI tract injury and associated pathogen-derived danger signals are critical to the propagation of the ‘‘cytokine storm’’ characteristic of acute GVHD (75). In humans, clinical studies clearly show that myeloablative conditioning, particularly TBI is a risk for acute GVHD (76, 77). Given this link between conditioning intensity and acute GVHD, non-myeloablative and reduced intensity conditioning regimens were developed by Storb and colleagues in dog models (78). The translation of these to humans were associated with reduced incidence of acute GVHD although later-onset acute GVHD, occurring after day 100 was noted (79).

### Antigen Presentation

In 1999, Shlomchik et al. demonstrated in preclinical mouse systems that recipient antigen presenting cells (APCs) were responsible for donor T cell activation and the induction of acute GVHD (80). They subsequently showed that although host APCs were much more potent, reconstituting donor hematopoietic APCs were necessary to invoke the full spectrum and severity of acute GVHD (81). They also demonstrated that these donor APCs could cross-present host antigens to induce chronic GVHD (82, 83). These hematopoietic (or professional) recipient or donor APCs were predominantly dendritic cells (DCs) (84, 85). Unexpectedly, Koyama et al. showed that non-hematopoietic recipient APCs exhibited a potent capacity to induce lethal acute GVHD (86) and consistent with this, depletion of recipient professional CD11c^+^ or CD11b^+^ APCs do not prevent GVHD (86, 87).

Subsequent mouse studies have demonstrated that intestine is a critical site for alloreactive T cell activation by APCs (86, 88, 89). Importantly, the pathogenic donor APCs in the colon are GM-CSF dependent, providing a potential therapeutic target [reviewed in (90)]. Intestinal epithelial cells highly express MHC class II and thereby regulate tolerance to intestinal commensals while inducing immunity against pathogens (91). Koyama et al. have demonstrated that prior to HCT, intestinal epithelial cells (IEC) express MHC class II in the ileum and can stimulate donor T cells and initiate acute GVHD, defining the lineage of the non-hematopoietic APC that initiates lethal GVHD (43). Both microbiota and conditioning invoke IL-12p40 dependent generation of interferon (IFN)-*γ* to mediate these effects by IEC (43). A translational clinical study has now commenced blocking IL-12p40 prior to conditioning in an attempt to prevent the initiation of MHC class II dependent GVHD within the GI tract (NCT04572815) (see also below).

Other mouse studies have been shown that intestine is a critical site for alloreactive T cell activation by APCs (86, 88). The α4β7 integrin-MAdCAM (mucosal addressin cell adhesion molecule) -1 pathway is critical for T cell homing to the intestine (88). Such a pathway found in mice has clinical potential for translation. Maraviroc is a small-molecule drug that block CCR5. However, addition of Maraviroc to standard GVHD prophylaxis composed of tacrolimus and methotrexate failed to reduce incidence of acute GVHD (92). Vedolizumab and Natalizumab, a humanized monoclonal antibody specifically target α4β7 integrin showed potentially promising results in phase I/II studies (93, 94), and these agents are currently tested in larger studies.

### Co-Stimulation

The activation, proliferation and differentiation of donor T cells requires recognition of alloantigen presented within MHC in the context of additional signals, usually a cognate costimulatory signal (characteristically CD40L – CD40 and CD28 – CD80/86 on the T cell and APC, respectively) and a differentiation signal in the form of cytokine as defined above. The recognition of the importance of cognate costimulatory signals has led to the investigation of the relative ability of inhibitory antibodies that block these pathways in preclinical models. Thus CTLA-4-Ig that inhibits CD80 and CD86, anti-CD28 and anti-CD40 have all been shown to attenuate GVHD in preclinical models (95–97). The clinical reagent abatacept (CTLA-4-Ig) has also shown promise in preventing acute GVHD in early phase clinical studies (98, 99). Additional pathways such as OX40L and ICOSL may also be clinically tractable [reviewed in (100)].

### Cytokines

Cytokines play a pivotal role in the pathogenesis of GVHD. Many inflammatory cytokines (e.g. IL-1, IL-6, TNF, HMGB1) are generated in response to chemoradiotherapy during conditioning and promote the activation of APCs. Other cytokines act in a costimulatory role to promote pathogenic T cell differentiation (e.g. IL-12, IL-6). Finally pivotal T cell derived cytokines (e.g. IFN*γ*, GM-CSF, IL-17) can in turn invoke target tissue apoptosis and secondary myeloid cell migration to amplify GVHD [reviewed in (100)]. Initial studies in experimental GVHD models suggested that cytotoxicity mediated by cytotoxic T lymphocytes (CTLs) have a central role in GVHD target tissue injury through the Fas/Fas ligand pathway and perforin/granzyme pathways (101–104). Subsequent studies demonstrated that inhibition of inflammatory cytokines such as TNF, IL-1 and IL-6 also ameliorated GVHD (74, 105–109). In 2002, Teshima et al. formally demonstrated that cytokines alone could generate the typical acute GVHD pathology in the absence of cognate cell-to-cell dependent cytotoxic mechanisms (110). These studies facilitated clinical trials of cytokine blockade for acute GVHD. However, individual cytokine blockade (e.g. TNF-α, IL-1, and IL-2) did not demonstrate significant benefits in randomized trials, suggesting considerable redundancy in these pathways and a likely requirement to inhibit multiple cytokines to gain clinical efficacy (111, 112). With this concept in mind, Ruxolitinib inhibits the signaling of multiple cytokines involved in the pathogenesis of experimental GVHD (113, 114) and a recent randomized study has demonstrated the efficacy of this agent in the treatment corticosteroid-refractory acute GVHD (115). This represents the first successful randomized study for the treatment of acute GVHD and highlights the successful translation of our understanding of the role of cytokines in GVHD from preclinical models. Ruxolitinib also targets Jak2, which relays signals for growth and differentiation of hematopoietic cells, in addition to Jak1, which relays inflammatory cytokines. Although it remains to be determined which pathway is critical for GVHD mitigation, animal studies suggest that neutrophils recruited to GI tract in response to bacterial translocation enhance intestinal GVHD *via* tissue damage (34). α1-Antitrypsin (ATT) inactivates serine proteases produced from neutrophils and macrophages and protect tissues from proteolytic degradation. Administration of AAT ameliorates murine acute GVHD *via* multiple mechanisms (116, 117). A phase 2 study of ATT shows promising results (118) and ATT is currently tested in larger studies.

### Tissue Homeostasis in GVHD

The pathophysiology of GVHD beyond donor effector T cells is now better understood. Damage to the intestine plays a central role in propagating a proinflammatory cytokine milieu and amplifying systemic GVHD. Indeed, intestinal GVHD is the major cause of non-relapse mortality after allogeneic HCT (75). Intestinal GVHD is characterized by severe villous atrophy and crypt degeneration; the latter has long been thought of as the cardinal and pathognomonic feature of intestinal GVHD (119–121). Recent data indicate that intestinal stem cells (ISCs) and their Paneth cell niche are targeted in GVHD, resulting in dysregulation of intestinal homeostasis and associated microbial ecology (122–124). Goblet cells are also significantly reduced in GVHD, resulting in disruption of inner mucus layer of the colon and increased bacterial translocation into colonic mucosa (125). In humans, reduced Paneth-cell numbers in duodenal biopsies and Goblet-cell numbers in colon biopsies correlate with the severity of GI-GVHD and transplant outcome (125, 126). Patients who undergo allogeneic HCT exhibit dysbiosis characterized by loss of diversity and expansion of potentially pathogenic bacteria (127–129). The microbiota and their metabolites shape the immune system and intestinal homeostasis (130). Lower microbial diversity and *Enterococcus* domination are associated with increased GVHD and poor survival across diverse ethnicity (40, 42). In addition, recent studies suggest an unexpected association between fungal and viral colonization and GVHD (39, 131). However, there are many open questions to be addressed in this field (132). What are the most important microbes that control transplant outcomes? Should we consider microbiota stewardship in addition to antibiotic stewardship in our transplant teams? Can we use interventions that modify the microbiome to improve transplant outcomes? What is the role of skin microbes in skin GVHD?

The sensitivity of target tissues to GVHD may be modulated by tissue-intrinsic resilience and homeostasis. Thus, integration of both immune tolerance and tissue tolerance could optimize GVHD treatment (133). In the 1990’s, Ferrara’s group proposed a concept of using cytokine shields to prevent target tissue damage in the GI tract. IL-11 and keratinocyte growth factor (KGF) protect gut injury from TBI and have anti-inflammatory properties. In mice, IL-11 and KGF ameliorated GVHD (134–136). However, a clinical trial of IL-11 was halted by unexpected severe side effects (137). This trial highlighted that clinical toxicity cannot always be estimated in mice and subsequent studies have also utilized primate models to study efficacy and toxicity (138). Protection of the ISC-niche and modification of the intestinal microbiota and metabolome to restore intestinal homeostasis may also represent a novel approach to modulate GVHD and also infection. In mice, IL-22 and R-Spondin are growth factors for ISCs that ameliorate GVHD in mice (122, 123, 139, 140). Glucagon-like-peptide-2 (GLP-2) is an enteroendocrine tissue hormone. Administration of GLP-2 promoted regeneration of ISCs and Paneth cells and restored intestinal homeostasis, resulting in amelioration of GVHD (141). IL-25 protects Goblet cells and also could improve transplant outcome (125). ISCs and Paneth cells express IFN-*γ* receptors. IFN-*γ* secreted by donor T cells induces ISC and Paneth cell death *in vitro* (142, 143). Ruxolotinib inhibits IFN-*γ* signaling and protects ISCs and Paneth cells (142, 143), which may be an additional mechanism of ruxolitinib’s action in intestinal GVHD (113, 144). Despite the promising mouse data, it remains to be elucidated whether modification of GVHD target tissue sensitivity can attenuate clinical GVHD.

Although most studies of tissue stem cell injury in GVHD have focused on the intestine, a recent study demonstrated that skin stem cells are injured in GVHD in association with impaired skin homeostasis (145). Topical corticosteroids suppressed skin inflammation but failed to protect skin stem cells and restore skin homeostasis. In contrast, topical ruxolitinib protected skin stem cells, resulting in restoration of hair regeneration and wound healing (145). These results in animals deserve further clinical scrutiny but will promote to open a new avenue for improved tissue homeostasis in GVHD beyond the GI tract.

## Chronic GVHD

Chronic GVHD, a pleiomorphic syndrome, is the major cause of nonrelapse mortality and severely impairs the quality of life in long-term survivors of allogeneic HCT. The highly variable clinical manifestations of chronic GVHD frequently involve the skin, liver, eyes, mouth, upper respiratory tract, esophagus, and less frequently serosal surfaces, lower gastrointestinal tract, female genitalia, and fascia (146). The biological mechanisms leading to chronic GVHD are not yet as well understood as those leading to acute GVHD, complicated by the fact that chronic GVHD can present with more heterogenous phenotypes than acute GVHD. Individual mouse models have dominant disease manifestations that typically involve a limited number of organs. The B6 into B10.BR model induces chronic GVHD primarily presenting as bronchiolitis obliterans (147). The B10.D2 into BALB/c model induces scleroderma as the primary manifestation (148). G-CSF-mobilized SCT (bothB6 into B6D2F1 and Balb/c to B6 mouse models) generate scleroderma, liver disease and Sjogren’s features (149). Using these preclinical models, significant progress has been made in the last decade and mouse models have demonstrated a critical role for donor Treg defects, germinal center B cell expansion and alloantibody secretion, and dysregulated Th17/Tc17 and T follicular helper (Tfh) differentiation in the development of chronic GVHD (150–157). Ibrutinib, an inhibitor of Bruton’s tyrosine kinase, has showed clinical efficacy in a phase II clinical trial and was approved for chronic GVHD, representing the first such agent (158). Treg are numerically decreased and dysfunctional in patients with chronic GVHD (159, 160). Low-dose IL-2 preferentially stimulates proliferation, function, and survival of Treg; low-dose IL-2 administration to patients has been shown expands Treg and ameliorates chronic GVHD in a proportion of patients (161, 162).　Efavakeukin-α, IL-2 mutein, is currently tested in a clinical trial. Ruxolitinib suppresses dysregulated inflammatory cytokine responses in chronic GVHD and is effective in patients with chronic GVHD (144); results of a prospective phase 3 trial of ruxolitinib for steroid refractory chronic GVHD are expected soon. Tfh and germinal center B cells (GCB) play a role in the development of chronic GVHD and bronchiolitis obliterans syndrome (152, 153, 163). The rho-associated coiled-coil kinase 2 (ROCK2) inhibitor, belumosudil (KD025), inhibits the differentiation of Th17 and Tfh together with GCB, and alloantibody generation (164). Syk inhibition induces apoptosis of activated B cells and ameliorates chronic GVHD (165, 166). Belumosudil and the Syk inhibitor Fostamatinib are currently being tested in clinical trials. Tissue fibrosis, the main manifestation of chronic GVHD, is characterized by increased deposition of collagen fibers secreted from activated fibroblasts in response to profibrotic cytokines such as TGF-β and PDGF-α secreted by CSF-1R-dependent macrophages (157, 167–172) [reviewed in (173)]. This pathological cascade of fibrosis defined in mice, has given rise to a number of new potential targets, including TGF-β, PDGF-α and CSF-1R; CSF-1R antibody axatilimab, which inhibits signaling through CSF-1 and IL-34, is currently undergoing assessment in clinical trials (NCT04710576).

## Conclusions

Mouse models of GVHD faithfully recapitulate the pathological lesions seen in clinical disease and allow the dissection of pathogenic *versus* protective immunological mechanisms of action and tissue resistance. While the ability to tightly control MHC and minor antigen barriers is a strength, the inbred nature of these systems may overlook variables present in outbred human populations (e.g. microbiota, age, obesity, prior therapy, comorbidities, conditioning, immune suppression). Some of these limitations can be overcome by more thorough study of these variables in mice (e.g. age, obesity, conditioning, concurrent immune suppression) and/or the use of non-human primates or dog models (especially pharmacological immune suppression). Additional limitations include the widespread use of cell lines to study graft-*versus*-leukemia effects and the lack of relevant models to study pathogen-specific immunity in the context of new therapies, at least until recently. Nevertheless, to date almost all effective therapeutic paradigms that are now established in humans have their genesis in animal models, suggesting that these systems will continue to provide valuable insights and therapeutic advances to the field. Importantly, it would seem critical that well-established preclinical systems are utilized to analyze the effects of various therapeutic interventions before they are translated into early phase clinical trials.

## Author Contributions

TT and GH, wrote the manuscript. All authors contributed to the article and approved the submitted version.

## Funding

TT is supported by JSPS KAKENHI (21H02944, 20K21610). GRH is supported by NIH R01 HL148164. The content is solely the responsibility of the authors and does not necessarily represent the official views of the NIH.

## Conflict of Interest

TT: Grants from Kyowa Kirin, Chugai, Sanofi, Astellas, TEIJIN PHARMA, Fuji Pharma, NIPPON SHINYAKU, Personal Fees from Novartis, Merck, Kyowa Kirin, Takeda, Pfizer, Bristol-Myers Squibb, Non-Financial Support from Janssen, Novartis.

GRH has consulted for Generon Corporation, NapaJen Pharma, Neoleukin Therapeutics, iTeos Therapeutics and has received research funding from Roche Pharmaceuticals, Compass Therapeutics, Syndax Pharmaceuticals, Applied Molecular Transport and iTeos Therapeutics.

## Publisher’s Note

All claims expressed in this article are solely those of the authors and do not necessarily represent those of their affiliated organizations, or those of the publisher, the editors and the reviewers. Any product that may be evaluated in this article, or claim that may be made by its manufacturer, is not guaranteed or endorsed by the publisher.
